# Effects of opioid blockade on taste perception across smoking status: an analysis of detection thresholds, intensity, and pleasantness

**DOI:** 10.1007/s00702-025-02937-9

**Published:** 2025-09-13

**Authors:** Justin J. Anker, Mustafa al’Absi

**Affiliations:** 1 Department of Psychiatry and Behavioral Sciences, University of Minnesota, Minneapolis, MN 55454 USA; 2 Department of Family Medicine and Biobehavioral Health, University of Minnesota Medical School, 1035 University Drive, Duluth, MN 55812 USA

**Keywords:** Taste perception, Smoking status, Suprathreshold intensity, Pleasantness, Endogenous opioid system, Naltrexone

## Abstract

The endogenous opioid system (EOS) is a neuromodulator of taste, and nicotine can modify both EOS signaling and taste perception. Understanding how nicotine interacts with EOS-driven taste perception is important for dietary guidance and smoking-cessation strategies. In this study, we tested whether opioid blockade with naltrexone vs. placebo differentially affects taste thresholds, intensity, and pleasantness in non-smokers, ad-lib smokers, and smokers in short-term withdrawal. A mixed factorial design was used; with drug (placebo vs. naltrexone) as a within-subject factor and smoking status (non-smoker, ad-lib smoker, withdrawal/abstaining) as a between-subjects factor. Each participant attended two sessions, receiving a placebo in one session and naltrexone in the other (counterbalanced). During each session participants completed (1) a sweet and bitter detection threshold test, and (2) suprathreshold intensity and pleasantness ratings for sweet, salty, sour, umami, and water. Results indicated that neither sweet nor bitter detection thresholds differed by drug or smoking groups. Among ad-lib smokers, however, suprathreshold intensity for sweet, salty, and sour tastes was significantly decreased under naltrexone vs. placebo, whereas pleasantness ratings remained unchanged. No drug effect on either intensity or pleasantness was observed in non-smokers and withdrawal smokers. The study’s results indicate a nicotine-related interaction with the EOS that reduces sensory gain but does not impact hedonic evaluation regarding taste perception

## Introduction

Taste perception is influenced by various physiological and neurological factors, including the endogenous opioid system (EOS). Early research demonstrated that μ-opioid–receptor activity amplifies the hedonic (“liking”) value of sweet and other highly palatable stimuli (Yeomans and Gray 1997; Drewnowski et al. 1995). For example, naltrexone-induced blockade of μ-opioid receptors reduces the pleas­antness, but not the perceived intensity of sweet solutions (Eikemo et al. 2016; Scińska et al. 2000), highlighting a partial dissociation between sensory gain and hedonic valuation.

Understanding how opioid pathways influence taste has clinical relevance, particularly for populations with chronically perturbed reward systems, such as smokers. Chronic smoking can alter taste perception (al’Absi et al. 2021; Anker et al. 2021; Koopmann et al. 2011; Le Strat and Le Foll 2011), possibly through peripheral sensory changes and central reward-pathway adaptations (Vennemann, Hummel, and Berger 2008). Smokers often display reduced taste sensitivity (Chéruel, Jarlier, and Sancho-Garnier 2017) and a preference for more intensely flavoured foods, perhaps compensating for nicotine-related sensory blunting (Ma and Lee 2023; Anker et al. 2021). After quitting smoking, taste sensitivity can recover within weeks, leading to changes in dietary choices as former smokers begin to experience enhanced taste (Chéruel, Jarlier, and Sancho-Garnier 2017).

Individuals who smoke without any restrictions may have different taste preferences and sensitivities compared to those who abstain from smoking for a short period. These differences may be related to the acute and chronic effects of to nicotine on neurochemical pathways, some of which overlap with opioid signaling (Balfour 2004). Indeed, nicotine’s ability to increase endogenous opioid release implicates the EOS in taste-driven reward among smokers (Kishioka et al. 2014). Additionally, the EOS interacts with stress systems (al’Absi 2018; al’Absi et al. 2020; Koob 2020; Slavich et al. 2014; Valentino and Van Bockstaele 2015), and is dysregulated by chronic tobacco use (al’Absi et al. 2008; Kishioka et al. 2014). This raises the possibility that nicotine influences opioid-related taste processing. One study found that smokers in short-term withdrawal consumed more calorie-dense “junk” food than non-smokers or smokers who continued to smoke, and naltrexone significantly attenuated this withdrawal-induced increase (Anker et al. 2021). However, it remains unclear whether opioid blockade similarly affects basic taste dimensions, such as detection thresholds, intensity, and hedonic ratings.

The present study examined how opioid blockade with naltrexone alters taste perception in non-smokers, ad-lib smokers, and smokers in short-term withdrawal. Our objectives were to determine whether opioid blockade (1) changes sweet and bitter detection thresholds, (2) affects suprathreshold intensity and pleasantness of sweet, salty, sour, umami (MSG), and water; and (3) interacts with smoking status.

## Methods

This study employed a mixed factorial design, incorporating one within-subject and one between-subject factor. Specifically, the within-subject factor was drug (placebo vs. naltrexone), while the between-subjects factor was smoking status (Non-smoker, Ad Lib, and Withdrawal/Abstain). Each participant attended two sessions, receiving a placebo in one session and naltrexone in the other, with the order counter balanced to minimize carry over effects.

### Participants

Participants were recruited based on their smoking status: Non-smokers: Individuals with no history of regular smoking or nicotine use. Ad Lib smokers: Individuals who smoked at least five cigarettes per day and were not required to abstain prior to testing. Withdrawal: Individuals who smoked regularly but abstained from smoking 12–24 h before each session. All participants were screened for normal taste function (i.e., no self-reported taste or oral health disorders) and general good health. Exclusion criteria included major medical conditions, pregnancy, or concurrent use of medications known to affect taste perception. Participants provided written informed consent, and the study protocol was approved by the University of Minnesota Institutional Review Board.

### Taste threshold testing (sweet, bitter)

#### Protocol overview

Detection thresholds for sweet (sucrose) and bitter (quinine) were determined with a modified staircase, two-cup forced-choice procedure. Solutions were presented in half-log concentration steps. Participants were instructed to taste each of the two cups (labeled “L” and “R”)—one containing plain water, the other containing the tastant—and identify which was the non-water cup. 

A correct response repeated the same concentration; a second correct response decreased the concentration, whereas an incorrect response increased it. Each switch from correct to incorrect (or vice versa) constituted a reversal.Participants rinsed and waited ~20s between trials to avoid habituation. After five reversals, the mean of the last few reversal concentrations defined the threshold.

### Suprathreshold intensity and pleasantness testing

Participants then evaluated five taste solutions-sweet (1.0 M sucrose), salty (1.0 M NaCl), sour (0.032 M citric acid), umami (0.0001 M MSG), and water (deionized control) - each solution presented in duplicate and in randomised order.

Participants sipped, held the solution until maximum intensity was perceived, and expectorated. Intensity was rated on the generalized Labeled Magnitude Scale (g-LMS), and pleasantness on a 10-point hedonic scale (“extreme dislike to extreme like”).

Ratings were recorded digitally. A deionized -water rinse and >60 s wait separated samples. Time-to-expectorate (TSE) was logged to gauge latency to maximum intensity.

### Statistical analysis

Repeated-measures ANOVAs were used to examine sweet and bitter thresholds ratings and suprathreshold intensity and pleasantness. Drug (placebo vs. naltrexone) was the within-subject factor and smoking status (non-smoker, ad-lib, withdrawal) was the between-subject factor. Significance was set at α = 0.05. Bonferroni corrections controlled the family-wise error rate. Homogeneity of variance (Levene’s test) and covariance (Box’s M) were checked; when violated, adjusted statistics (e.g., Greenhouse-Geisser correction) were applied.

## Results

### Sample characteristics

Table 1 summarizes participant demographic. Mean age was comparable among groups (*p* =.569) and body- mass index (BMI) also showed no between-group differences (*p* =.195). Years of education differed by smoking status (*p* <.001): non-smokers reported more years (15.1) than both the adlib (12.8) and abstain (13.3) participants. Gender and distribution and race did not differ significantly across groups.

### Taste threshold results (sweet, bitter)

**Fig. 1:**
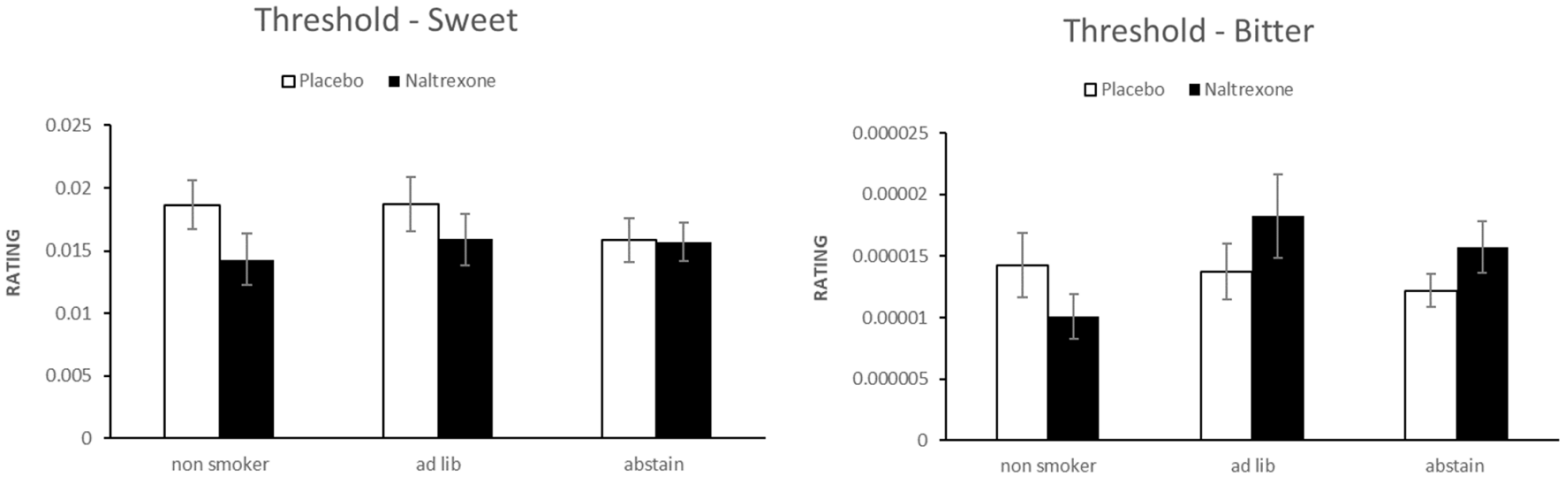
Sweet (left panel) and bitter (right panel) taste threshold ratings by smoking status and drug condition. Error bars represent standard errors of the mean

Figure 1 presents mean +SEM thresholds for sweet (left) and bitter (right).A repeated-measures ANOVA revealed no main effects of Drug (placebo vs naltrexone) or Smoking Status, and no Drug × Smoking Status interaction for either taste. 

### Suprathreshold intensity and pleasantness results

#### Taste intensity

**Fig. 2:**
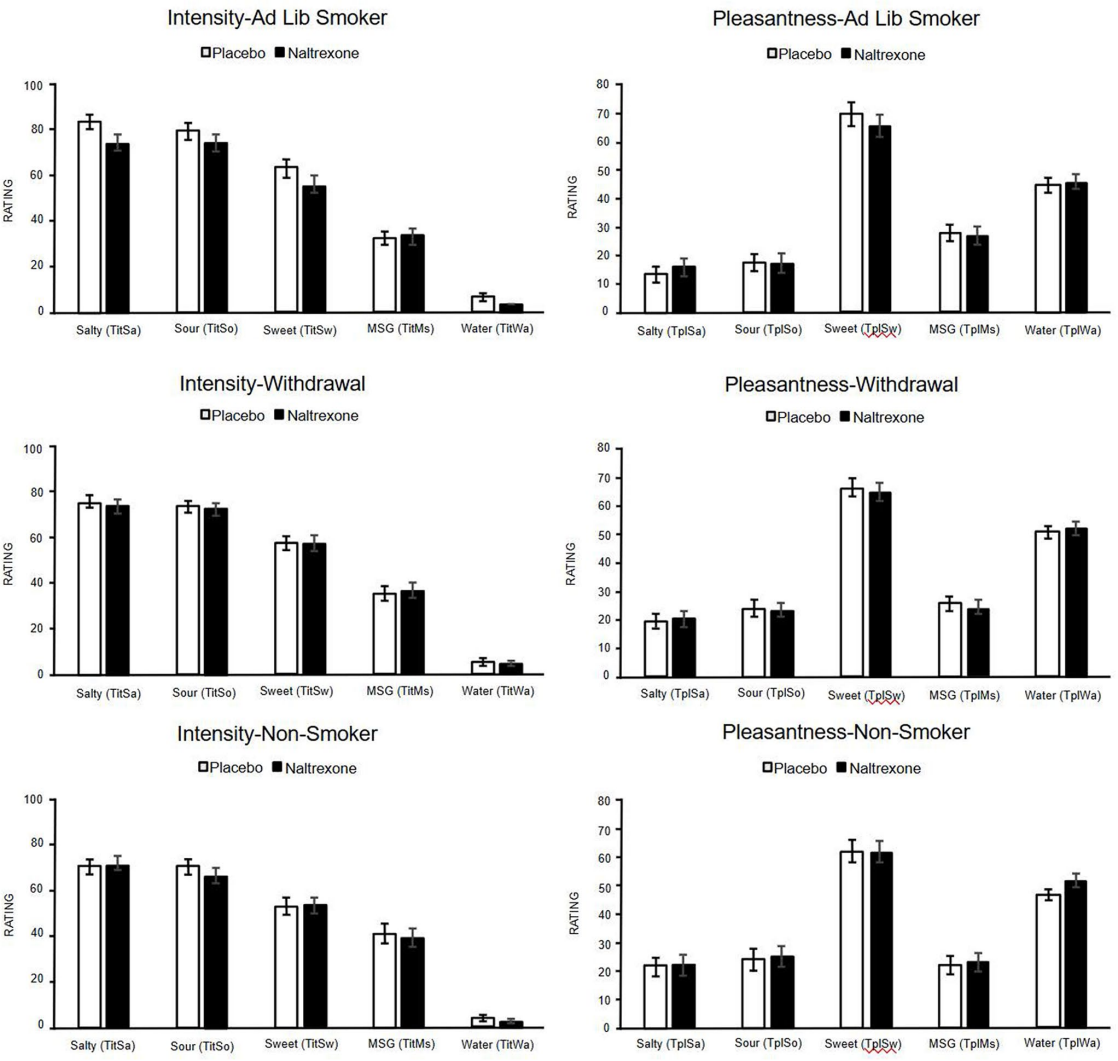
Effects of Naltrexone on taste intensity (left panels) and pleasantness (right panels) across smoking groups. Error bars represent standard errors of the mean

Figure 2 displays the intensity ratings (left panels). Sweet intensity did not significantly differ by drug, smoking status, or their interaction. Exploratory pairwise comparisons showed that ad-lib smokers rated sweet intensity higher under placebo than naltrexone (*p* =.031), whereas the other two groups showed no difference.

For salty intensity, a significant drug effect was found (F,₁,₁₄₃ = 7.104, p =.009): ratings were higher under placebo than naltrexone. A significant drug × smoking status interaction (F₂,₁₄₃ = 4.696, p =.011)   indicated that the difference between placebo and naltrexone was driven mainly by the ad-lib group: ad-lib smokers showed a pronounced drop from 82.9 (placebo) to 72.4 (naltrexone; *p* <.001). Non-smokers and withdrawal smokers did not differ significantly between drug conditions. Under placebo, ad-lib smokers’ salt intensity ratings exceeded those of non-smokers and nearly exceeded those of abstainers, while under naltrexone groups were comparable.

For sour intensity, a significant drug effect was found (F(1, 143) = 7.101, *p* =.009) without interaction, indicating that, on average, participants rated sour taste lower under naltrexone than placebo. Ad-lib smokers showed a small but significant drop (~ 6 points) under naltrexone (*p* =.033). Non-smokers and withdrawal smokers did not exhibit significant changes from placebo to naltrexone. Water and MSG intensities showed no significant main effects or interactions (all p >.05). 

#### Taste pleasantness

Figure 2 shows pleasantness ratings (right panels). Salty and sweet pleasantness were unchanged by drug, smoking status, or their interaction. Water pleasantness increased under naltrexone (F₁,₁₄₃ = 5.113, p =.025) and this was driven by non-smokers. Pairwise tests indicated a ~6-point rise for non-smokers following naltrexone compared to placebo (*p*=0.009). Sour and MSG pleasantness were unaffected by drug, smoking status, or their interaction.

## Discussion

This study examined the effects of opioid receptor blockade (naltrexone vs. placebo) on taste perception in non-smokers, ad-lib smokers, and short-term withdrawal smokers. Our objectives were to determine whether opioid antagonism (1) alters detection thresholds for sweet and bitter tastes, (2) modifies the intensity and pleasantness ratings across five taste modalities, and (3) whether any effects vary by smoking status.

The results showed that naltrexone did not change the tresholds for sweet or bitter which aligns with prior work showing that opioid blockade has minimal effects on basic taste sensitivity (Doty et al. 1994; Arbisi et al. 1999; Parker et al. 1992). Although non-smokers showed a nominal decrease in sweet threshold under naltrexone, their ability to perceive taste at threshold levels remained relatively unaffected by acute EOS disruption.

At suprathreshold levels, ad-lib smokers demonstrated selective reductions in the intensity of sweet, salty, and sour tastes, while experiencing no change in pleasantness when under naltrexone. This diverges from laboratory studies in non-smokers, where naltrexone lowered pleasantness while leaving perceived intensity unchanged (Drewnowski et al. 1995; Eikemo et al. 2016). One possible explanation for this difference is the partial dissociation between “how strong” a taste feels and “how good” it tastes. μ-Opioid receptors in the brainstem and anterior insula amplify incoming gustatory signal, whereas hedonic appraisal involves downstream processing within the mesolimbic dopamine circuits and orbitofrontal cortex (Berridge and Kringelbach 2015). Blocking μ-receptors could dampen sensory gain (lowering intensity) without diminishing pleasantness, especially if nicotine continues to activate dopaminergic reward pathways. Active nicotine use can elevate dopaminergic and opioid signaling (Benowitz 2008), potentially rendering taste pathways more sensitive to blockade. Consistent with this interpretation, hedonic ratings remained stable across drug conditions, except for a small increase in water pleasantness among non-smokers.

The impact of smoking status on how opioid blockade affected taste was notable; ad-lib smokers—those with ongoing nicotine exposure—were most susceptible to reduced taste intensity, whereas withdrawal smokers resembled non-smokers. This pattern highlights the EOS's contribution to nicotine-induced reward and suggests that the interaction between nicotine and endogenous opioids may be strongest during active smoking (Xue and Domino 2008; Kishioka et al. 2014). Importantly, the preservation of pleasantness alongside reduced intensity indicates that nicotine enhances opioid-driven sensory gain more than it does hedonic valuation. These results extend prior evidence linking naltrexone to decreased intake of palatable foods (Anker et al. 2021) by demonstrating that opioid modulation spans multiple taste modalities, not just sweetness. 

The broader implications of these results indicate that chronic nicotine exposure also disrupts stress pathways that intersect with the EOS (Koob and Volkow 2010; al’Absi et al. 2020). Stress can blunt sweet-taste intensity, and cortisol correlates with changes in the perception of salt and sour tastes (al’Absi et al. 2012). Future work should test whether stress further magnifies—or mitigates—the nicotine × naltrexone interaction observed here.

Limitations of the current study include the relatively brief withdrawal interval, which may not fully capture the effects of prolonged abstinence; potential demographic or education-level differences; and the inherent constraints of a within-subject design that does not assess long-term changes with extended naltrexone use or sustained smoking cessation. Future investigations might extend the withdrawal period or measure biochemical markers (e.g., cotinine) to better characterize the trajectory of taste-related reward recovery post-cessation. Additionally, exploring how modulators such as stress, mood, or individual variations in opioid receptor polymorphisms, could deepen understanding of how these factors shape taste perception during nicotine use and/or abstinence.

In conclusion, our findings demonstrate that opioid blockade selectively attenuated suprathreshold taste intensity but did not affect pleasantness among ad-lib smokers. This nicotine-dependent reduction in sensory gain suggests that targeting intensity loss, rather than liking, may be a valuable approach for dietary guidance and opioid-based adjuncts in smoking-cessation programs.

**Table 1:** Demographic and baseline characteristics by smoking group

	Non-Smoker	Ad Lib	Abstain	*p*-value
Age (years)	36.97 (1.98)	34.26 (2.12)	34.59 (1.65)	0.569
BMI (kg/m²)	26.07 (0.95)	28.55 (1.02)	26.83 (0.80)	0.195
Education (years)	15.09 (0.43)*	12.82 (0.45)	13.29 (0.35)	< 0.001
Gender (% female)	48.7%	38.2%	42.9%	0.662
Race (% caucasian)	73.7%	71.9%	70.9%	0.487

Note: Values are Mean (Standard Error) or percentage of the study sample. BMI = body mass index. *Non-smokers have significantly higher education levels than both Ad Lib and Abstain groups (post hoc tests, *p* <.01). No significant differences were observed among groups for gender, ethnicity, marital status, or race (*p* >.05)
